# Virucidal Action Mechanism of Alcohol and Divalent Cations Against Human Adenovirus

**DOI:** 10.3389/fmolb.2020.570914

**Published:** 2020-12-17

**Authors:** Natalia Martín-González, Leonam Vieira Gonçalves, Gabriela N. Condezo, Carmen San Martín, María Rubiano, Ian Fallis, Joseph R. Rubino, M. Khalid Ijaz, Jean-Yves Maillard, Pedro J. De Pablo

**Affiliations:** ^1^Department of Condensed Matter Physics, Universidad Autónoma de Madrid, Madrid, Spain; ^2^School of Pharmacy and Pharmaceutical Sciences, Cardiff University, Cardiff, Wales, United Kingdom; ^3^Department of Macromolecular Structures, Centro Nacional de Biotecnología-Consejo Superior de Investigaciones Científicas, Madrid, Spain; ^4^School of Chemistry, Cardiff University, Cardiff, United Kingdom; ^5^Center of Innovation, Reckitt Benckiser Inc., Montvale, NJ, United States

**Keywords:** virus damage, biocides, virus mechanics, AFM, adenovirus

## Abstract

Hygiene and disinfection practices play an important role at preventing spread of viral infections in household, industrial and clinical settings. Although formulations based on >70% ethanol are virucidal, there is a currently a need to reformulate products with much lower alcohol concentrations. It has been reported that zinc can increase the virucidal activity of alcohols, although the reasons for such potentiation is unclear. One approach in developing virucidal formulations is to understand the mechanisms of action of active ingredients and formulation excipients. Here, we investigated the virucidal activity of alcohol (40% w/v) and zinc sulfate (0.1% w/v) combinations and their impact on a human adenovirus (HAdV) using, nucleic acid integrity assays, atomic force microscopy (AFM) and transmission electron microscopy (TEM). We observed no difference in virucidal activity (5 log_10_ reduction in 60 min) against between an ethanol only based formulation and a formulation combining ethanol and zinc salt. Furthermore, TEM imaging showed that the ethanol only formulation produced gross capsid damage, whilst zinc-based formulation or formulation combining both ethanol and zinc did not affect HAdV DNA. Unexpectedly, the addition of nickel salt (5 mM NiCl_2_) to the ethanol-zinc formulation contributed to a weakening of the capsid and alteration of the capsid mechanics exemplified by AFM imaging, together with structural capsid damage. The addition of zinc sulfate to the ethanol formulation did not add the formulation efficacy, but the unexpected mechanistic synergy between NiCl_2_ and the ethanol formulation opens an interesting perspective for the possible potentiation of an alcohol-based formulation. Furthermore, we show that AFM can be an important tool for understanding the mechanistic impact of virucidal formulation.

## Introduction

Understanding the mode of action of a biocidal products has been shown to be relevant for establishing scientific principles, improvement of biocidal products as well as usage optimization, and combatting emerging resistance by target microorganism (Myers, 2008; Condell et al., 2012). Biocides are multi-target antimicrobial agents with broad spectrum of action. Understanding their interactions with microbial targets, here viruses, informs our knowledge of mechanisms contributing to viral inactivation or viral resistance mechanisms, and contributes to improving efficacy through formulation design and better usage recommendation of the product in practice (Russell, 2003). Non-enveloped viruses offer less targets for virucidal action compared to enveloped ones. These targets comprise mainly the capsid, viral encoded receptor binding proteins and viral genomes (Maillard, 2001; Myers, 2008). The mechanisms of virucidal action against non-enveloped viruses remain poorly studied.

The increased use of alcohols over other antimicrobials can be attributed to their rapid and broad-spectrum antimicrobial activity against bacteria, viruses and fungi (Mcdonnell and Russell, 1999; Guthery et al., 2005). Alcohols show substantial virucidal activity against enveloped viruses as compared to non-enveloped ones, which suggests that the viral lipid envelope is a potential target (van Engelenburg et al., 2002). Alcohol in concentrations between 59 and 90% (w/v) show a fast acting and broad-spectrum of antimicrobial action (Mcdonnell and Russell, 1999; Macinga et al., 2008; Alhmidi et al., 2017), but persistence of efficacy is relatively low as alcohols evaporate quickly, and their residual activity after short periods of time is thus compromised (Rutala and Weber, 2014; Alhmidi et al., 2017). Moreover, usage of 60–90% (w/v) alcohol in surface and hand disinfectants can be problematic mostly due to increased flammability, toxicity and generation of high amounts of volatile organic compounds (VOCs) affecting user safety (Kramer et al., 2006). VOCs generated by household products containing aforementioned alcohol concentrations may have short and long-term adverse health effects on animal and human in indoor environments including sensory irritation, allergies, asthma and leukemia (Suchomel et al., 2009). The design of formulations that accommodate a decrease in alcohol concentration while retaining an appropriate virucidal activity is thus essential. Alcohols in synergistic combination with other antimicrobials (e.g., metals) are being studied not only to address those issues but to confer higher efficacy and persistence to a biocidal product as well (Gaonkar et al., 2006). The usage of zinc salts and oxides as antimicrobial agents is still limited. The main usage of zinc is as preservative in combination with other active ingredients in biocidal products used as pesticide in agriculture (Rajasekaran et al., 2016), as hand and skin antisepsis in household and healthcare products (Guthery et al., 2005; Gonçalves et al., 2012), and as antifouling agent in paints for metal surface treatment (Ytreberg et al., 2010). The combination of alcohol and zinc salts has not been widely reported, but the use of zinc pyrithione has shown some benefit on formulation antimicrobial activity and persistence (Guthery et al., 2005). Usage of other zinc salts such as zinc sulfate (as used in the present study) in combination with alcohol has not been reported commercially or academically yet.

In this context, the main goal of this study was to analyse the capacity of ethanol/zinc salt combination to present virucidal activity against non-enveloped viruses with identification of possible targets leading to a better understanding of the mechanism(s) of action. The influence of such combination against mammalian virus (adenovirus) capsid susceptibility to mechanical stress and virus integrity was verified through a novel nanoindentation analysis by Atomic Force Microscopy (AFM). Furthermore, virucidal efficacy testing alongside viral purification and subsequent DNA extraction after exposure to formulations was performed in order to elucidate whether viral nucleic acid is a potential target for the formulation system under study.

Overall, this study sought to confirm the potentiation of the virucidal activity of formulated ethanol with divalent cations, and to understand the mechanisms of action for such activity against human adenovirus.

## Materials and Methods

### Formulations

The formulations tested where obtained from Reckitt Benckiser and their basic composition are described in Table 1. Excipients (non-active ingredients) are not disclosed due to proprietary issues. The virucidal efficacy of unformulated biocides are most commonly studied, and investigation of formulation is rarely reported in the literature. This study looked at the effect of the formulation (RB-Full) and controls consisted of formulated ethanol (RB-ethanol) and zinc (RB-zinc).

**Table 1:** Formulations studied and their composition.

**Formulation**	**Composition**
RB-Full	40% (w/v) ethanol + 0.1% (w/v) zinc sulfate + excipients; pH 10.5
RB-Ethanol	40% (w/v) ethanol + excipients; pH 10.5
RB-Zinc	0.1% (w/v) zinc sulfate + excipients; pH 10.5
RB-Control	Excipients; pH 10.5

### Propagation and Purification of Human Adenovirus Type 2 and 5

The human adenovirus type 5 variant FC31-attP (HAdV5/attP) was used for the AFM and TEM investigations. HAdV5/attP is structurally similar to the wild type human adenovirus type 5, but its genome contains some deletions for genes involved in replicative cycle control (E1 region) and host immune system evasion (E3 region) (Alba et al., 2007). It also expresses green fluorescent protein (GFP) to facilitate tracking of virus amplification kinetics and titration. The wild type human adenovirus type 2 (HAdV2) is used in virucidal standard efficacy tests.

HAdV5/attP and HAdV2 were propagated in HEK293 and HeLA cells, respectively. Infected cells were harvested 36 h (HAdV5/attP) or 3 days (HAdV2) post-infection and viral particles were purified by ultracentrifugation in cesium chloride (CsCl) gradients (Condezo et al., 2015), after centrifugation of the freeze-thaw disrupted cells to remove cell debris. TD1X Buffer (137 mM NaCl, 5.1 mM KCl, 0.7 mM Na_2_.HPO_4_.7H_2_O and 25 mM Tris base at pH 7.4) was used to prepare CsCl gradients. Ultracentrifugation was conducted at 219,000 g (LE-80K Ultracentrifuge, Beckman Coulter) for 90 min at 18°C in 1.25 and 1.40 g/ml CsCl step gradient. Following a first centrifugation step, viral bands were extracted, deposited in a tube containing 1.31 g mL^−1^ CsCl and centrifuged at 219,000 g for 18 h to form a continuous gradient. Virus particles extracted from the gradient were desalted through column chromatography (Bio-Rad 10 DC, UK), eluted with HBS (20 mM Hepes, 150 mM NaCl, pH 7.8) and stored in aliquots with 10% glycerol at −80°C.

HAdV2 infectious concentration was quantified using the Spearman-Kaeber method 6 and 7 days post-infection (19) and concentration expressed as PFU ml^−1^. HAdV5/attP was quantified by spectrophotometry (Hitachi Model F-2500 FL) using the hexon fluorescence emission spectra. Briefly, 0.15 mL samples of diluted viral preparations in sealed quartz cuvettes were excited at 285 nm, and emission spectra were measured from 310 to 375 nm using excitation and emission slit widths of 10 nm. The spectra were corrected by subtraction of the buffer spectrum. The maximum emission intensity for each spectrum was found at 333 nm and recorded. The concentration expressed as viral particles mL^−1^ was determined from a calibration curve calculated from a virus preparation with a known concentration.

### Determination of Virucidal Activity

The BS EN 14476 quantitative virucidal suspension test (BS EN 14476:2013+A2, 2019) in clean condition, i.e., with sterile hard water as the only interfering substance and without organic load, was carried out against HAdV2. Briefly, 500 μl of test viruses were prepared by adding 340 μl of HAdV2 stock suspension (10^9^ PFU/ml) to 160 μl of distilled water. One hundred mocroliter of the test HAdV2 was then added to 900 μl of formulation. After 60 min, 100 μl of test viral suspension was added to a Microspin S-200 HR size-exclusion chromatography column (GE, UK) to quench the activity of the formulation activity and to reduce host cell cytotoxicity. Then, 100 μl of the mixture were serially diluted in 900 μl purified water (1:10 serial dilutions). Each dilution (100 μl) was added to wells of 24 tissue culture plates containing with 70–80% confluence HeLa cells monolayer. The infective virus concentration was determined by Spearman-Kaeber method, 6 and 7 days post-infection (Flint and American Society for Microbiology, 2009). Reduction in infectivity is expressed as PFU/ml which relate to the number of infectious particles.

### Viral DNA Extraction, Quantification, Analysis of DNA Damage

The DNA analysis post viral treatment method described in Maillard et al. (1995) was adapted for HAdV2. Twenty microliter of purified HAdV2suspension (10^9^ PFU/ml) was added to 80 μl of each formulation for 2 h at 25°C. Control consisted of replacing the suspension with PBS. HAdV2 DNA was extracted and purified using a high pure viral nucleic acid kit (Roche, Switzerland). Briefly, 200 μl of binding buffer supplemented with 50 μl proteinase K and poly(A) (Roche, Switzerland) was added to the treated virus suspension and incubated at 72°C for 15 min. The mixture was then added to a high pure filter column and centrifuged three times at 8,000 g for 1 min, with flow-through being discarded after each centrifugation cycle. The column was then washed twice with 450 μl of wash buffer (Roche, Switzerland). Elution of HAdV2 dsDNA occurred by adding 40 μl of elution buffer and centrifugation at 13,000 g for 1 min. Viral DNA was stored at −20°C until analysis by electrophoresis. Then 150 ng of purified viral dsDNA was mixed with 5 μl of gel loading buffer and loaded on 1.2% (w/v) agarose gel in TBE buffer with SYBR Safe DNA gel stain (ThermoFischer, United Kingdom). The lambda DNA/HindIII marker size profile was used.

Purified HAdV2 dsDNA was digested separately by SmaI and AatII restriction enzymes (Thermo Scientific, Germany) in order to evaluate more specifically potential damage to viral nucleic acid caused by formulations (see Table 1 for details). After digestion, electrophoresis was performed on a 2% (w/v) agarose gel in TBE buffer at 100 V for 1 h and 30 min. Viral dsDNA bands were visualized as described earlier.

### Electron Microscopy

Five μL of a HAdV5/attP preparation containing 1 × 10^12^ viral particles/ml previous dialyzed against HBS during 1 h at 4°C. This concentration refers to physical particles. The virus sample was diluted in HBS with and without 5 mM NiCl_2_ and incubated on glow discharged collodion/carbon coated grids for 5 min, blotted and incubated with 45 μL of formulation for different times and conditions. Grids were washed with HBS (500 μL), stained with 2% (w/v) uranyl acetate for 30 s and examined in a JEOL JEM 1,230 transmission electron microscope at 100 kV.

### Atomic Force Microscopy

Purified HAdV5/attP previous dialyzed against HBS during 1 h at 4°C was diluted in HBS with 5 mM NiCl_2_ to a concentration of 1.5 × 10^12^ viral particles/ml. Twenty microliter of virus suspension were then deposited on freshly cleaved mica and incubated for 20 min at 4°C. After washing the sample with HBS-5 mM NiCl_2_ to remove the non-adsorbed particles, 180 μl of formulation were added for a set exposure time and then washed again with HBS-5 mM NiCl_2_ buffer and immersed in 500 μl of HBS with 5 mM NiCl_2_ (Supplementary Figure 1). Short exposure times of 0.5, 1, 2.5, 5, and 10 min were chosen to verify whether the structural integrity of the majority of the viral particles was maintained or not. Measurements were performed using an AFM (Nanotec Electrónica S.L., Madrid, Spain) operating in jumping plus mode (Ortega-Esteban et al., 2012) in liquid milieu with a force of 70 pN. Cantilevers (RC800PSA, Olympus, Tokyo, Japan) with nominal spring constants of 0.05 N/m were used and calibrated using Sader's method (Sader et al., 1999). AFM nanoindentation (force vs. z-piezo displacement; FZ) experiments were performed on viral particles to obtain their mechanical properties (Supplementary Figure 3) at a certain incubation time.

## Results

### Virucidal Activity of Formulations

**Figure 1:**
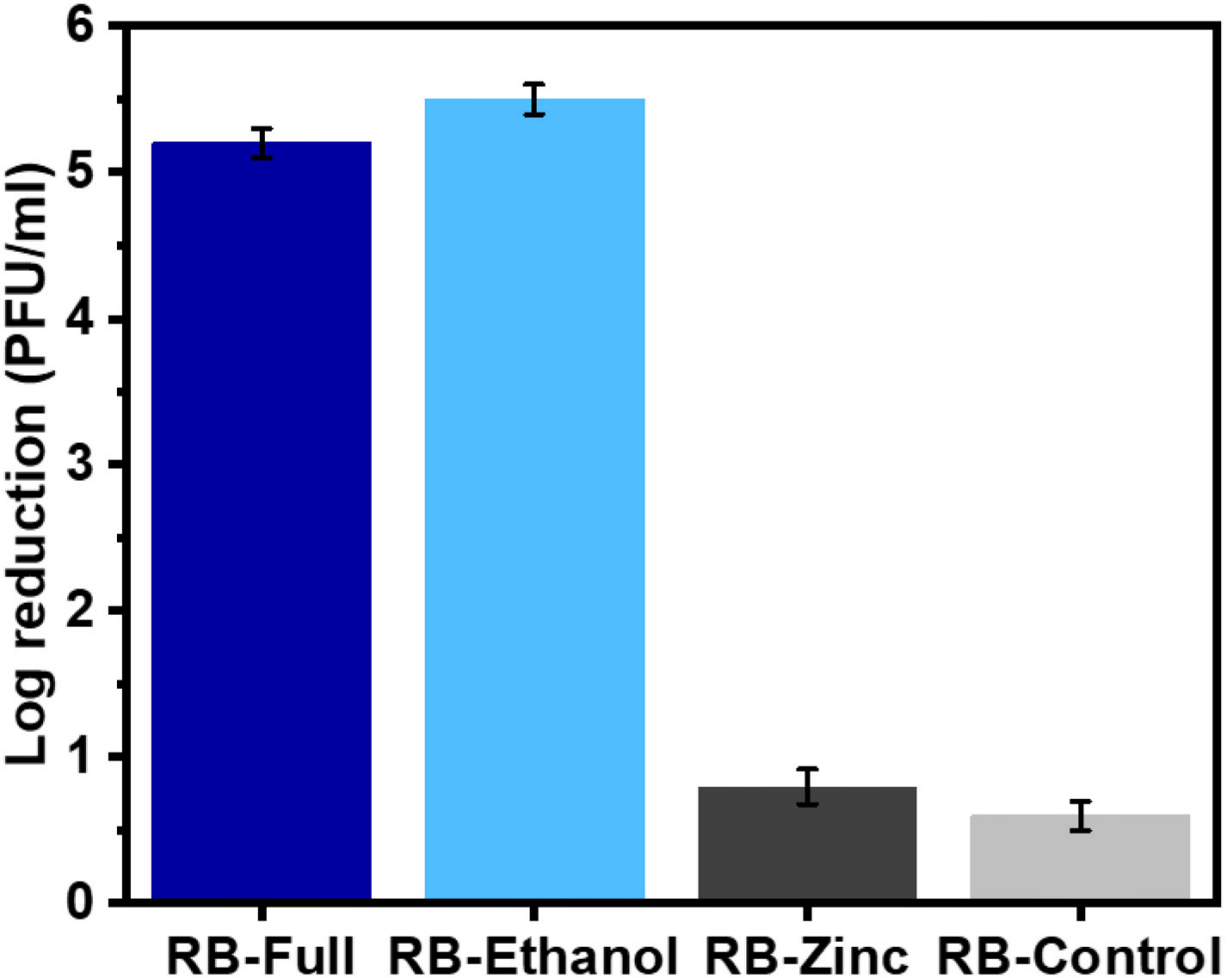
Efficacy of the different formulations against HAdV2 using the EN14776 suspension test. Test conditions: 60 min contact time, hard water, no organic load.

Following the use of the BS EN14476 protocol, the virucidal activity of the different formulations after 60 min exposure in hard water but with no organic load showed that the full formulation and the formulated ethanol (RB-Full and RB-Ethanol) (Figure 1) showed a significant reduction in PFU/ml compared to the formulated zinc or the control containing excipients only (*p* < 0.05). The formulation control (RB-Control) which contained excipients only did not show any reduction in HAdV2 infectivity and the lack of virus inactivation was comparable to RB-Zinc (Figure 1). Furthermore, there was no difference in activity between RB-Full and RB-Ethanol.

### DNA Damage Analysis

**Figure 2:**
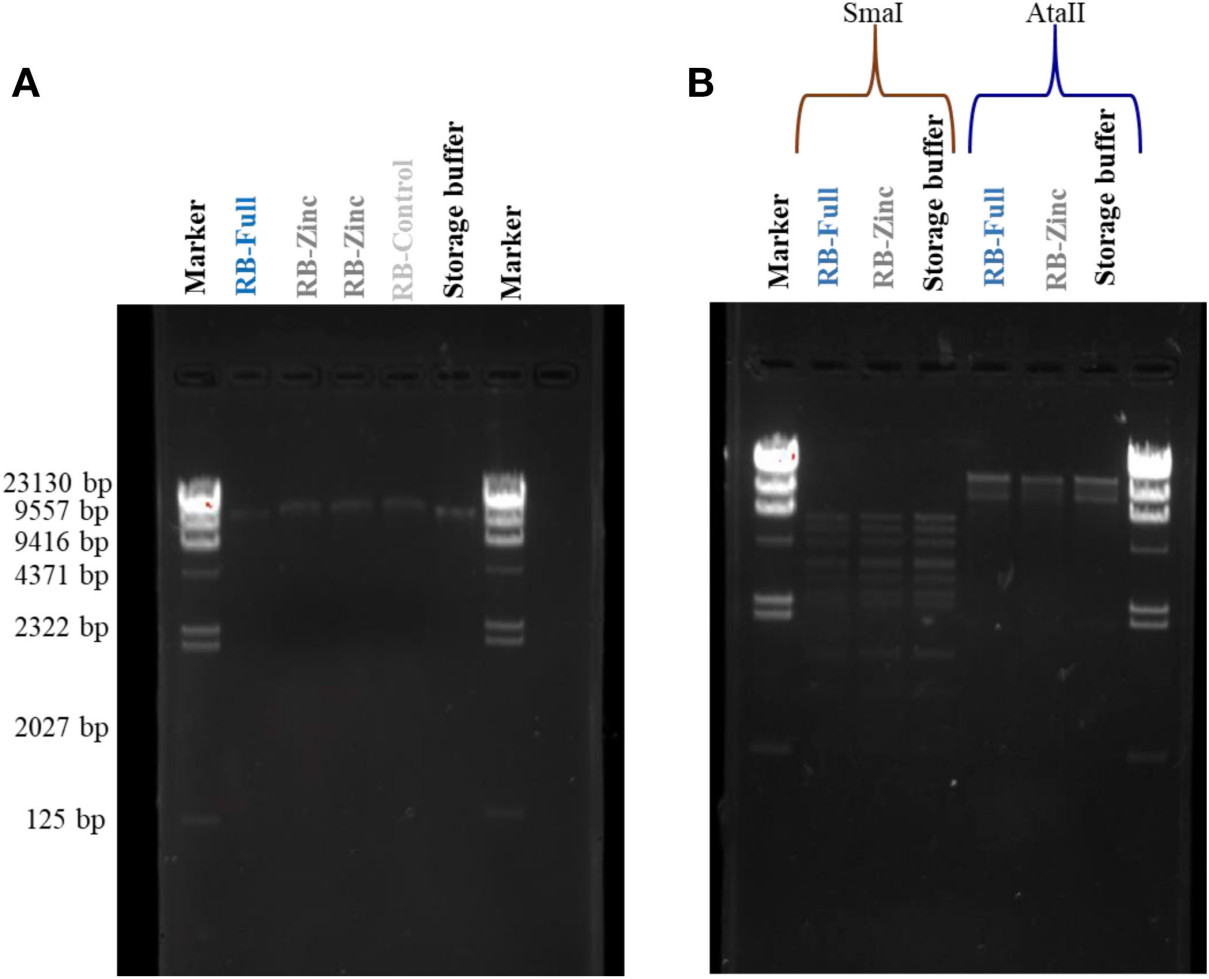
HAdV2 DNA damage analysis after exposure to formulations. **(A)** HAdV2 DNA bands extracted after exposure to different formulations; **(B)** HAdV2 DNA bands obtained after exposure to formulations and digested separately by SmaI and AatII restriction enzymes (see text).

Potential damage to viral DNA was evaluated for HAdV2. There was no apparent DNA damage following virus exposure to any of the formulations. Severe damage associated with random multiple breaks in DNA (Maillard et al., 1995; Elnifro et al., 2000) would take the form of a smear (Figure 2A). In addition, the use of restriction enzymes, which would indicate random DNA breaks, did not show any differences in DNA band pattern following virus exposure to different formulations (Figure 2B).

### Morphological Analysis

**Figure 3:**
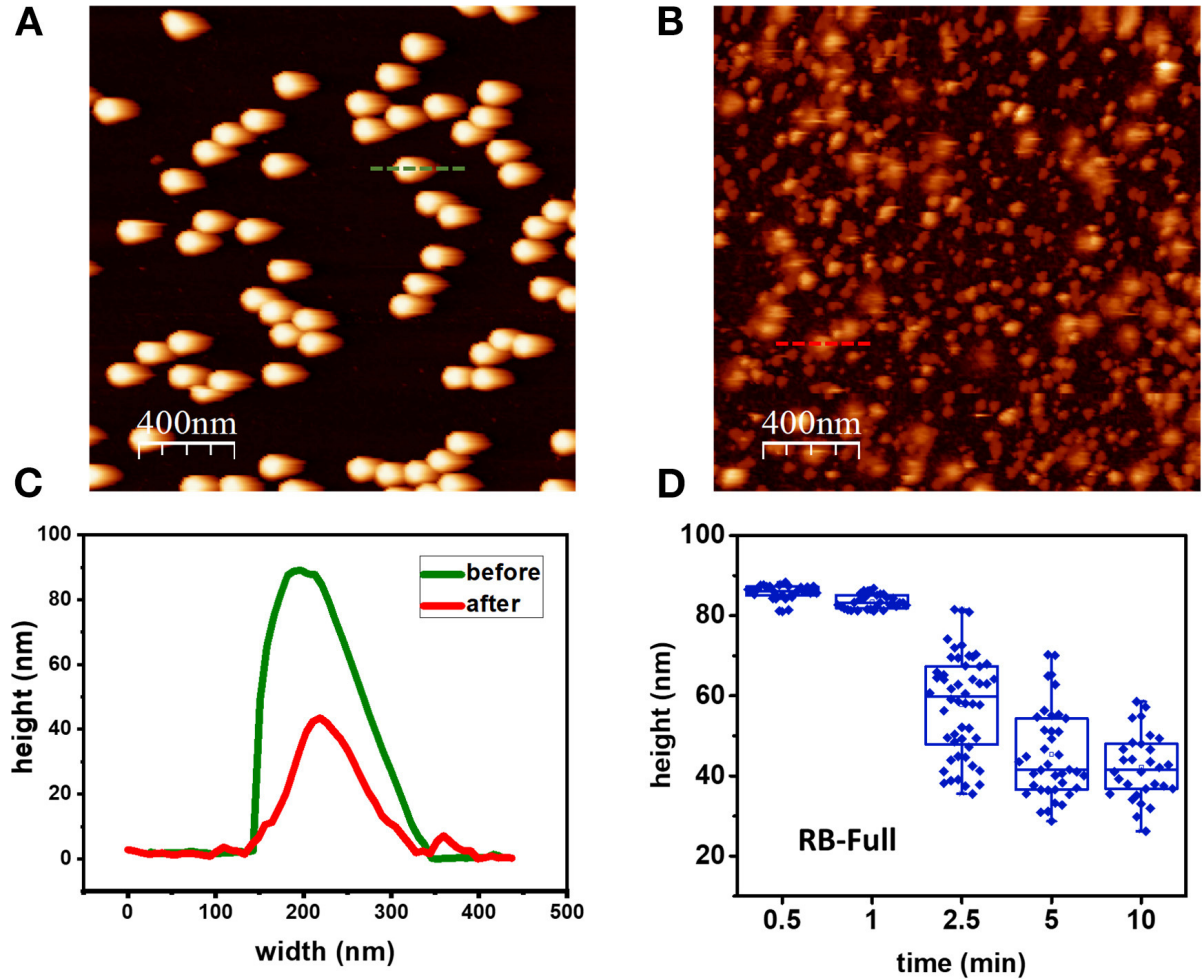
Morphology changes induced by formulation RB-Full. **(A)** Control: AFM image taken before treatment. **(B)** AFM image taken after 5 min contact with RB-Full. **(C)** Profiles of one viral particle and before (green line) and after (red line) treatment with RB-Full. **(D)** Box plot showing the evolution of particle height as a function of treatment time with RB-Full.

**Figure 4:**
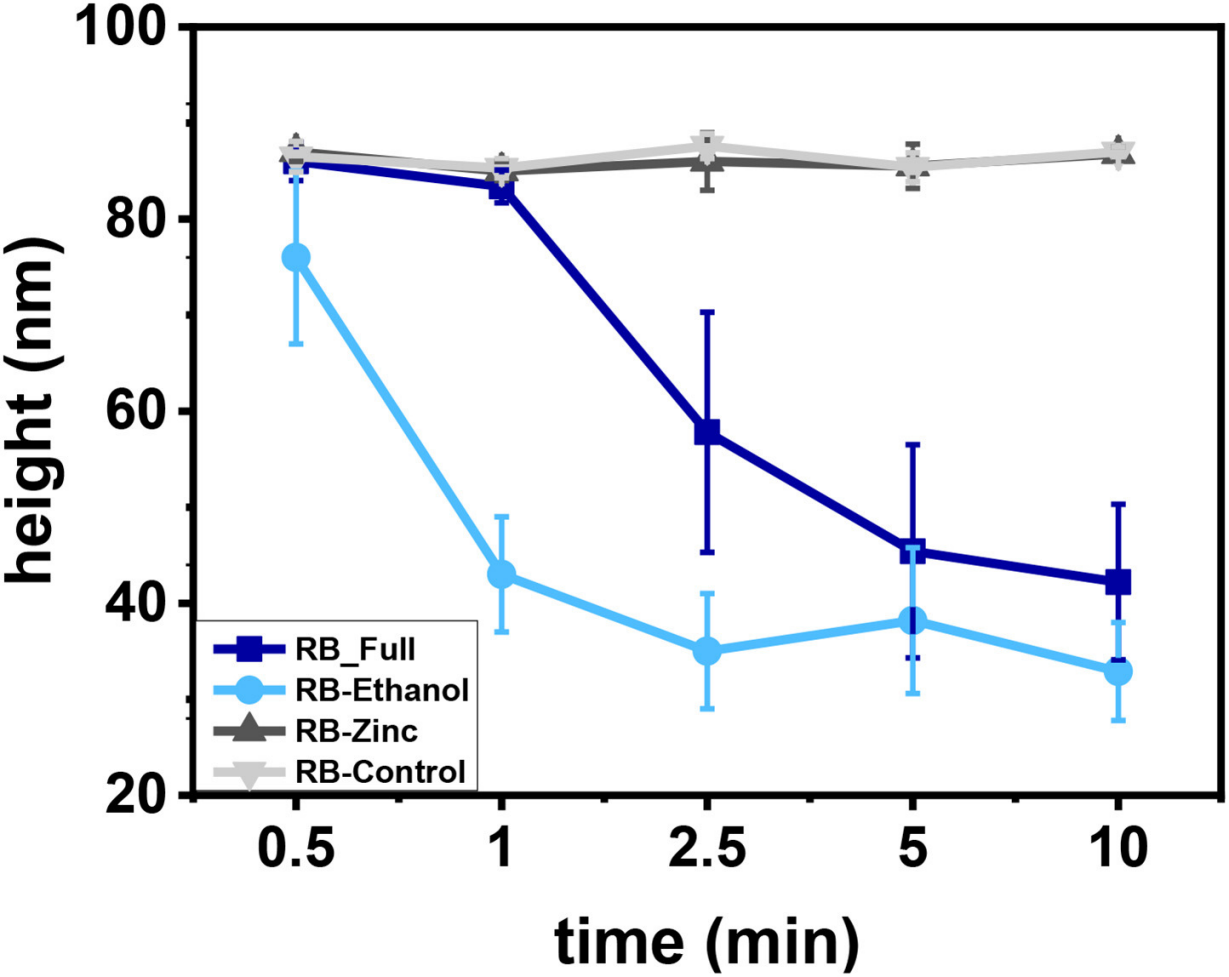
Particle height evolution after treatment with the different formulations as a function of time. Height average values and standard deviation are plotted. The *N* = 50 for all the formulations and contact times.

The effect of the different formulations in HAdV particle morphology was analyzed using AFM imaging in liquids and TEM. HAdV5/attP particles were imaged after exposure to the different formulations at various incubation times. Figure 3A shows a typical AFM topography of a random population of HAdV5/attP particles obtained in liquid condition before treatment. The topographical profile of one of the particles, marked by a green line in Figure 3C indicated that the particle size is 86 nm, in agreement with the nominal diameter of a HAdV virion (San Martín, 2012). This revealed that the interaction with the mica surface or AFM imaging did not alter the particle structure. The impact of the different formulations on the particle morphology following different incubation times was then measured. In Figure 3B we showed an example of HAdV5/attP incubation in RB-Full for 5 min, that resulted in drastic morphological alterations. The topographical profile (red line in Figure 3C) demonstrated that the height for the particle taken as an example decreased to 45 nm (a 50% reduction for this particular virion). Analysis of a particle height dataset showed decreases with increasing RB-Full exposure time up to 52% after 10 min (Figure 3D), indicative of capsid disruption or collapse. Moreover, RB-Ethanol significantly induced morphological changes in HAdV5/attP (Figure 4 and Supplementary Figure 2). This is supported by a decreasing of the height with increasing incubation time. RB-Full decreased the height of the particle by 52% after 5 min of exposure time, whilst RB-ethanol decreased virus particle height by 62% after 2.5 min (Figure 4). RB-Zinc and RB-Control did not produce any height reduction in viral particles, regardless of the exposure time (Figure 4). RB-Full which contains both ethanol and zinc seemed to have a lesser effect on the integrity of the viral particles causing less damage when compared to the formulation containing only ethanol (RB-Ethanol).

**Figure 5:**
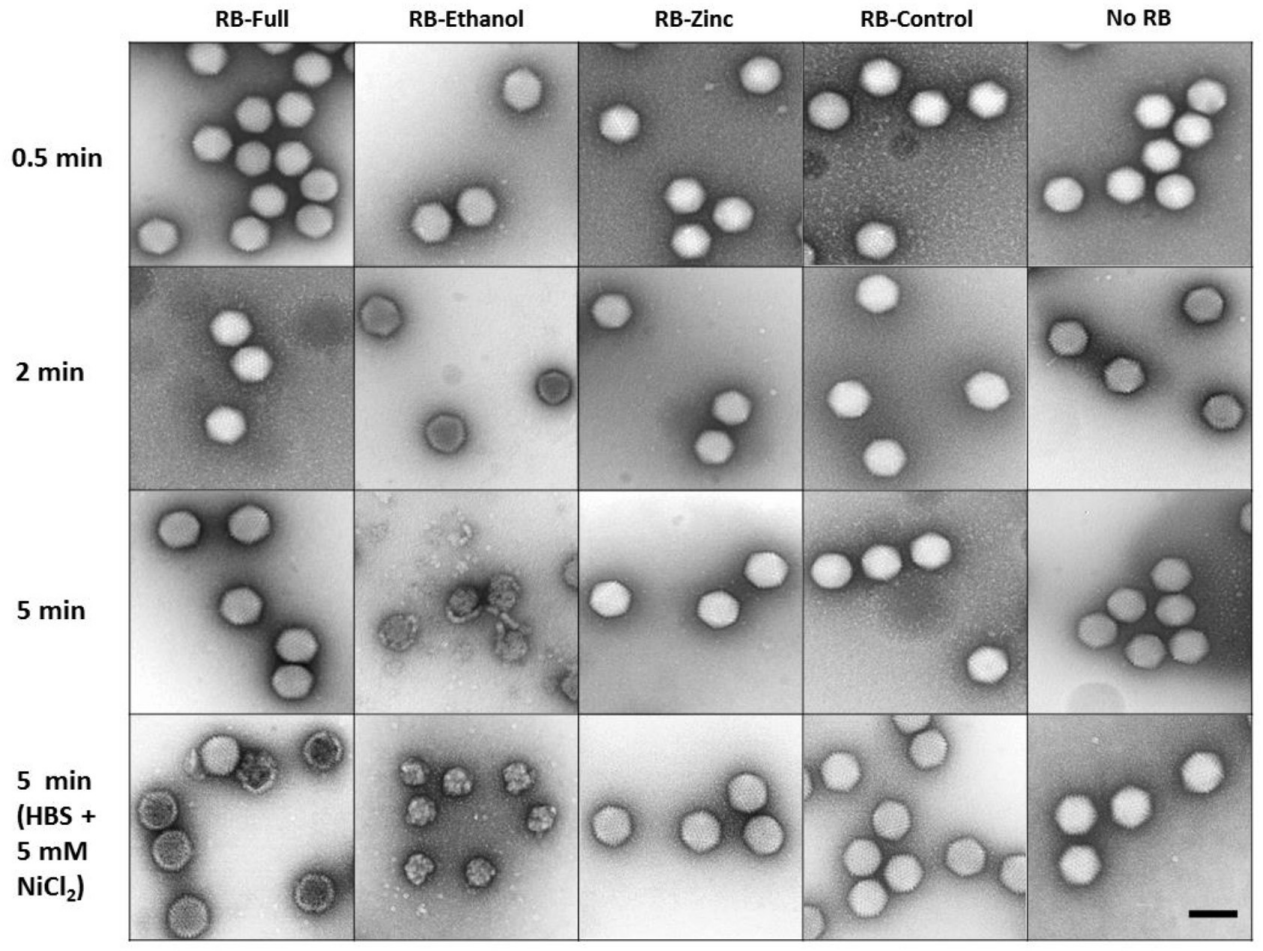
Negative staining TEM imaging of HAdV particles treated with the indicated formulations for different time lengths and buffer conditions. Scale bar 100 nm.

On the other hand, TEM images only revealed damage when treated with RB-Ethanol after 5 min of incubation (Figure 5). The other formulations did not reveal any gross morphological changes in HAdV5/attP particles independently of the contact time when only HBS was present (Figure 5). The apparent discrepancy between the AFM and TEM results with RB-Full formulation may originate from the differences in the experimental setup between the two imaging techniques. In particular, AFM imaging of HAdV particles adsorbed to mica requires the presence of Ni^+2^ ions in the buffer (Ortega-Esteban et al., 2013). We hypothesized the possibility of a synergistic effect between RB-Full and nickel salt used for the AFM assays. To verify this hypothesis, we repeated the TEM experiments adding Ni_2_Cl to the HBS buffer at the same concentration used in the AFM protocol. This addition showed some damaged viral particles after 5 min of incubation by TEM (Figure 5 and Supplementary Figure 2), which still do not suffice to provide the average height showed in AFM (Figure 4, dark blue). The excess of disrupted particles found in AFM can be ascribed to the fact that virus specimens are typically subjected to forces of ~100 pN during imaging. It is known that such low force can disrupt previously weakened virus structures (Ortega-Esteban et al., 2015). In the present case, it is likely that virus particles are deteriorated by the combination of RB-Full and NiCl_2_ and destroyed during AFM imaging. Therefore, a combination of RB-Full with 5 mM NiCl_2_ is responsible for the structural damage observed using both AFM and TEM.

### Mechanical Properties

**Figure 6:**
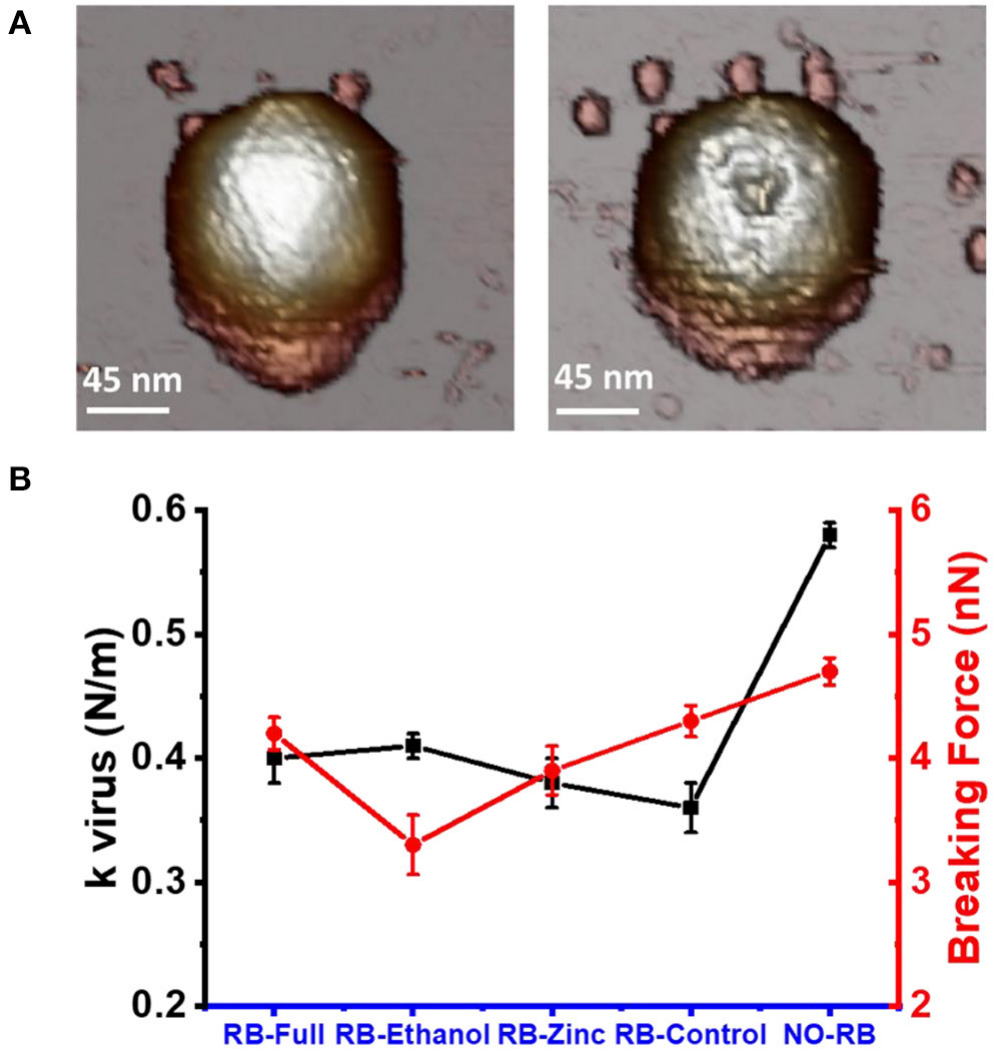
Changes in mechanical properties of HAdV5/attp capsids following exposure to formulations. **(A)** AFM image taken before and after a nanoindentation experiment. **(B)** Black symbols represent the spring constant (measured as k virus N/m) and red symbols represent the breaking force (measured as nN) for the three formulations tested and the untreated control. Values plotted are listed in Table 2.

Changes in the mechanical properties of the HAdV5/attP virions were measured as stiffness (spring constant) and breaking force (Supplementary Figure 3). An incubation time of 0.5 min with the formulations diluted 1:1 in Milli-Q H_2_O was chosen to have structurally intact viral particles that can be analyzed mechanically. Using this contact time, we ensured there was no reduction in height of HAdV-5/attP caused by the formulation. Intact viral particles exhibited a linear behavior until the elastic limit was reached breaking the particle (Figure 6A). The particle spring constant was significantly (ANOVA, *p* < 0.05) lower in samples exposed to the formulations when compared to untreated control, showing that particles became softer when incubated with the formulations (Table 2 and Figure 6B). However, no significant variation was found between the effect of the four formulations, as indicated by a Kolmoworov-Smirnov test of the measurements. This observation indicates that the excipients and highly basic pH present in all formulations tested alter HAdV particle stiffness. In contrast, exposure of HAdV5/attP to RB-Ethanol significantly decreased the breaking force indicating a weakening of the viral capsid when comparing with the other formulations. The maximum required force to break the particle is decreased in this case showing that the viral particles are more brittle (i.e., for the same deformation the force to break the particle is lower).

**Table 2:** Mechanical properties of HAdV5/attP particles after treatment with the indicated formulations for 0.5 min.

**Formulation**	**Spring constant (N/m)**	**Breaking Force (nN)**	***N***
RB-Full	0.40 ± 0.02	4.2 ± 0.1	38
RB-Ethanol	0.41 ± 0.02	3.3 ± 0.2	36
RB-Zinc	0.38 ± 0.02	4.0 ± 0.2	37
RB-Control	0.36 ± 0.02	4.3 ± 0.1	31
NO RB	0.58 ± 0.01	4.7 ± 0.1	100

*N, number of virions observed*.

## Discussion

Biocidal products play an important role for the control or elimination of microbial contamination in a wide range of settings. Some microorganisms, and notably non-enveloped viruses are more challenging to inactivate than enveloped ones due to the lack of a lipid bilayer envelope as a potential target (Ijaz and Rubino, 2008). The development of synergistic formulations based on combining active ingredients or/and selecting appropriate formulation excipients is important as this can lead to significant increase in disinfection efficacy while reducing toxicity and improving surface compatibility. Overall, there is little understanding of the mechanisms underlying virucidal activity of alcohol and metal ions in a given formulation. In this study, we aimed to produce a better insight of the use of zinc salt to potentiate the virucidal activity of a ethanol formulation against a non-enveloped virus. In our experimental set up we decided to use ethanol and the different control in a formulation as this would represent better the use of a final disinfectant product. Besides the impact of a formulation on biocidal activity is rarely investigated, hindering the application of findings to the efficacy of a final product in practice.

The RB-Full [0% (w/v) ethanol + 0.1% (w/v) zinc salt; pH 10.5] and the RB-Ethanol [40% (w/v) ethanol + excipients; pH 10.5] formulations were virucidal against HAdV2 (> 5 log_10_ reduction) within 60 min at room temperature and without organic load. RB-Zinc [0.1% (w/v) zinc sulfate + excipients; pH 10.5] on the control (excipients only) were not. Unformulated ethanol is considered to have a rapid virucidal activity (within 2 min) against adenovirus type 2 at concentrations between 55 and 85%. For adenovirus type 5, the reported effective concentration of unformulated ethanol ranges between 45 and 95% (Kampf, 2018). No gross structural damage of the virions was observed by TEM using RB-Full which contrasted with the damaging effect of RB-Ethanol. Although TEM observations cannot be directly correlated to the level virucidal activity, the mechanisms of virions inactivation between the 2 formulations could be different, since both RB-Full and RB-Ethanol showed the same virucidal efficacy. These findings also questioned the role of zinc in the full formulation. We originally hypothesize that sufficient damage to the viral capsid imparted, for example, by ethanol would allow zinc penetration and its interaction with viral DNA. Zinc has been shown to interact directly with dsDNA (Evilevitch et al., 2011). Free zinc ions (Zn^2+^) have been shown to be able to bind to DNA and change its secondary structure mainly through interactions with DNA phosphate sugar backbone as well as guanine and cytosine (Aich et al., 1999). A high ratio of Zn^2+^ to DNA has also been shown to destabilize the DNA double helix decreasing its melting temperatures (Souza et al., 2000; Labiuk et al., 2003). In addition, the viral genome can impact on capsid mechanics (Labiuk et al., 2001; Ivanovska et al., 2007). However, we did not observe that zinc caused any damage to viral dsDNA.

Although no major structural damage of the virions was observed by TEM using RB-Full, the addition of Ni^2+^ produced gross alterations of the viral particles. Such gross damage was confirmed with AFM suggesting capsid weakening with increased incubation time. We hypothesize that NiCl_2_ was necessary to observe gross viral particle alteration when combined to the RB-Full formulation. The use of NiCl_2_ in the AFM protocol was necessary because divalent ions are required for virus adsorption on mica. The potential mechanistic synergistic effect combining the RB-Full formulation with NiCl_2_ was not expected and opens an interesting avenue for further formulation optimization. The mechanical properties of the viral capsid such as stiffness and capacity to withstand pressure such as DNA packing and cell entry process, and extracellular conditions including osmotic pressure, desiccation and pH, plays a significant role for their viability (Carrasco et al., 2011; Greber, 2016). Research on the mechanical properties of adenovirus exposed to the full formulation in the presence of NiCl_2_ resulted in a reduction of the capsid rigidity. The observed lower breaking force in this study indicates that the virus would be more susceptible to environment chemical and mechanical stress (Hernando-Pérez et al., 2014). RB-Ethanol with NiCl_2_ affected the ability of the virus to withstand pressure rather than its stiffness, ultimately decreasing the viral capsid ability to withstand mechanical stress (Hernando-Pérez et al., 2014). The mechanical properties of the virions were measured on intact particles in order to avoid the influence of structural defects (cracks, vacancies, etc.). In this way, we were able to identify subtle changes on the capsomer bonds induced by the formulations. From our data (Figure 6) differences in the mechanical alterations of viruses between formulations were not extensive. However, we observed that all the formulations tested produced changes in the virus mechanics which contrasts with untreated viruses (Figure 6B).

Although it is tempting to explain virucidal activity with the observed damage to the viral particles, there is no direct correlation between gross viral particle alteration and infectivity assays.

## Conclusion

The need for biocidal product manufacturers to decrease alcohol concentration for environmental, regulatory and commercial reasons, while maintaining virucidal activity is challenging. We hypothesized that the addition of zinc might potentiate a low (non virucidal) concentration of ethanol (here 40%). We combined different techniques to investigate the interplay between the infectivity, integrity and mechanical effects of ethanol and zinc salt as active ingredients against HAdV. We observed that HAdV particles were inactivated (5 log_10_ reduction in PFU/ml) when exposed to the RB-full and RB-Ethanol for 60 min in clean conditions, although only the formulated ethanol produced gross morphological change to the viral particles after 5 min exposure. It was interesting to observe that the presence of 5 mM NiCl_2_ contributed to the structural damage imparted by the full formulation. AFM confirmed that capsid topography reduced considerable when virus was exposed to the full formulation in te presence of NiCl_2_. Although the addition of zinc did not seem to improve the efficacy of the formulation, the addition of NiCl_2_ contributing to a weakening of the viral capsid offers an interesting avenue to pursue.

The use of AFM was instrumental in making such observations and as such, AFM has shown to be an important tool for understanding the mechanistic impact of virucidal formulation.

## Data Availability Statement

The original contributions presented in the study are included in the article/Supplementary Material, further inquiries can be directed to the corresponding author/s.

## Author Contributions

NM-G and LV peformed AFM and virucide experiments, respectivelly, and wrote the paper. GNC peformed TEM experiments. MR, IF, JR, and MI prepared virucides. CSM, J-YM, and PD designed research and wrote the paper. All authors contributed to the article and approved the submitted version.

## Conflict of Interest

JR and MI are employed by the company Reckitt Benckiser Inc. The remaining authors declare that the research was conducted in the absence of any commercial or financial relationships that could be construed as a potential conflict of interest.
