# Supplementary material for: Virucidal Action Mechanism of Alcohol and Divalent Cations Against Human Adenovirus
Source: Front Mol Biosci. 2020 Dec 17;7:570914. doi: 10.3389/fmolb.2020.570914 (PMC7773831; doi:10.3389/fmolb.2020.570914)

SUPLEMENTARY INFORMATION

Supplementary figures

Figure S1. Experimental procedure. First, a HAdV drop of 20 µl is deposited in a mica muscovita surface. After waiting 20 minutes the sample is washed with HBS buffer (HBS+5mM $NiCl_2$. As a control, AFM images are taken before the formulation is added. By fixing the incubation time required the formulation is removed and a buffer drop of 500 ul is added at the same time. Finally, AFM images are taken to perform the experiments.

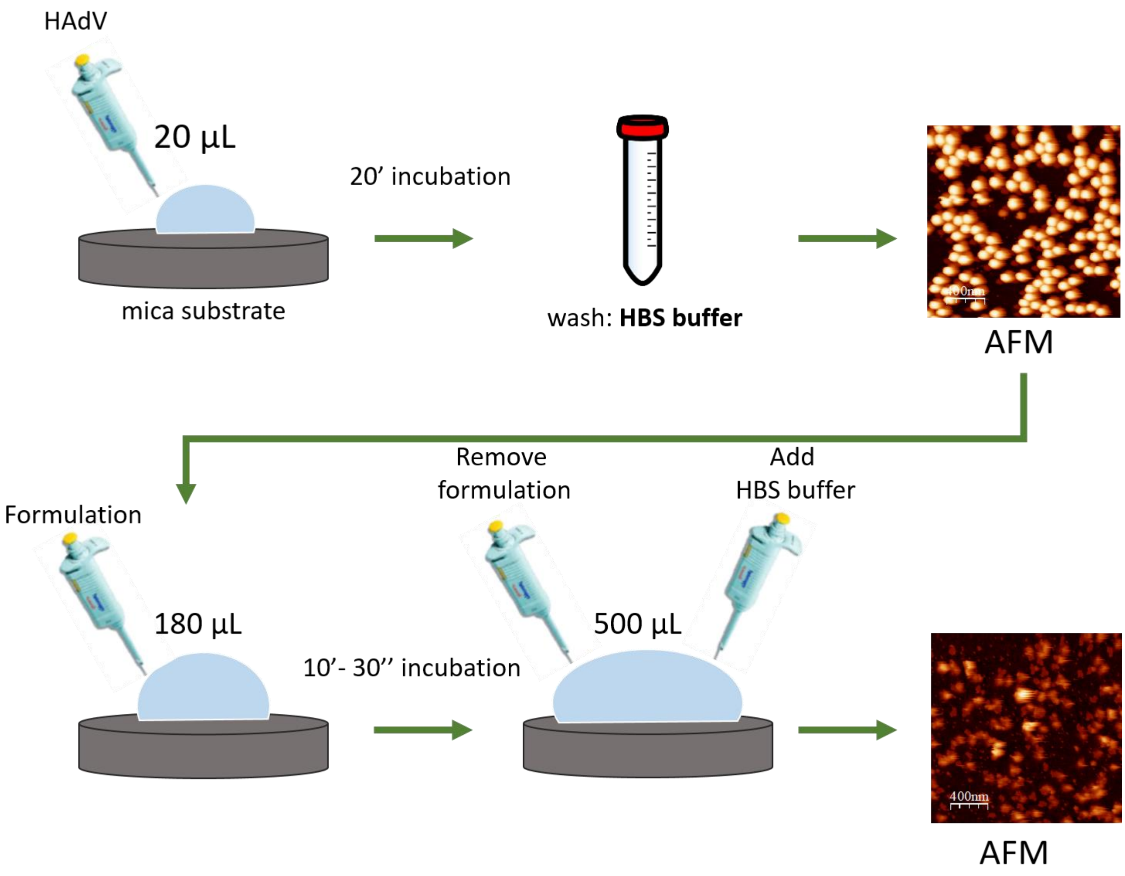

Figure S2. Morphology changes induced by all the formulations. (a) AFM images taken before and after the virucide treatment at the five different contact times used. (b) Box plot showing the evolution of particle height as a function of treatment time with RB-Full, RB-Zinc and RB-Control.

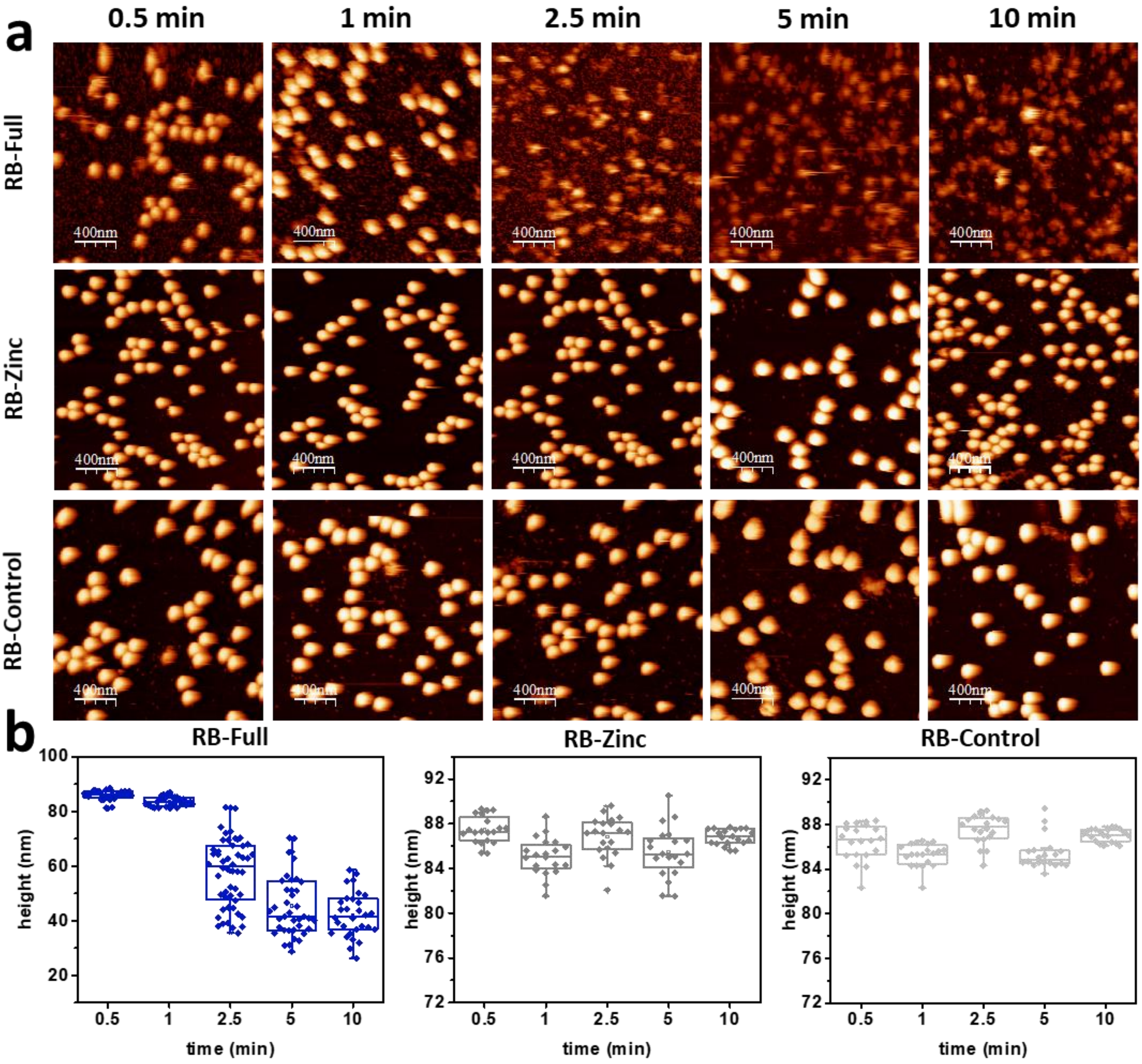

Figure S3. Force curve on an adenovirus particle**.** (a) Topography images of a human adenovirus particle before (left) and after (right) perform an indentation. (b) Indentation curve computed from a force distance curve (FZ). The spring constant and the breaking force are obtained from this curve.

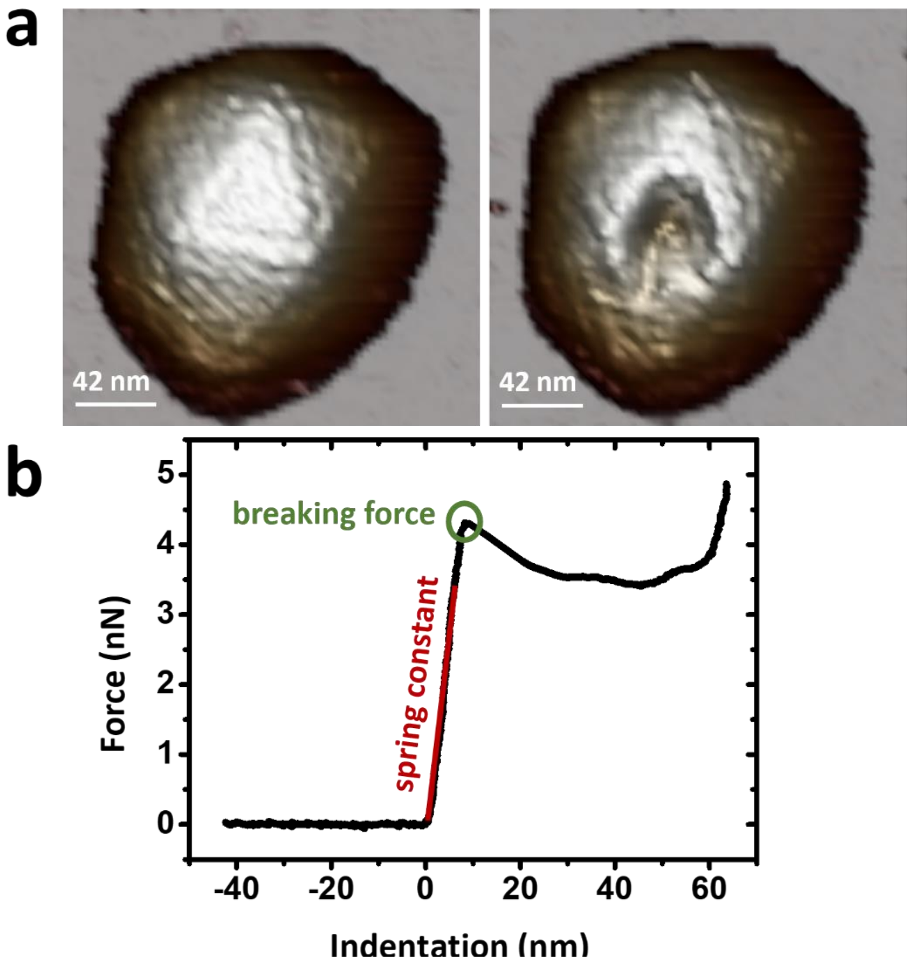

Supplement: Supplementary file 1 [file Data_Sheet_1.pdf]
